# Network Destabilization in Aging: Mitochondrial Dysfunction, Nutrient Sensing, and Chronic Inflammation as Interconnected Drivers

**DOI:** 10.3390/molecules31132317

**Published:** 2026-07-01

**Authors:** Wojciech Rzeski

**Affiliations:** Department of Functional Anatomy and Cytobiology, Institute of Biological Sciences, Maria Curie-Skłodowska University, Akademicka 19, 20-033 Lublin, Poland; wojciech.rzeski@mail.umcs.pl

**Keywords:** aging, integrative biology, network medicine, mitochondria, mitophagy, NAD^+^ metabolism, chronic inflammation, multimorbidity

## Abstract

Aging is the dominant risk factor for most chronic diseases, yet the mechanisms driving this relationship remain poorly integrated across biological scales. Existing frameworks have catalogued key hallmarks of aging but do not explain how these processes converge to produce organism-level decline and multimorbidity. A systems-level framework is introduced in which aging is conceptualized as progressive destabilization of interacting regulatory networks. Mitochondrial quality control, nutrient-sensing pathways, and chronic inflammatory signaling form a putative high-centrality network core: mitochondria coordinate redox balance, bioenergetics, and transcriptional adaptation, while NAD^+^-dependent signaling and NLRP3 inflammasome activation propagate perturbations across regulatory layers. This architecture provides a mechanistic basis for the convergence of neurodegenerative, cardiovascular, metabolic, and oncological phenotypes as emergent consequences of shared network instability. Reframing the hallmarks as coupled network nodes shifts the explanatory focus from isolated mechanisms to system-level resilience and non-linear dynamics. This narrative and conceptual review integrates evidence across mitochondrial biology, metabolic signaling, and inflammatory pathways to develop these arguments, with explicit acknowledgment that the proposed framework is hypothesis-generating rather than formally validated. Interventions targeting high-centrality nodes, including mTOR modulation, NAD^+^ restoration, mitophagy activation, and anti-inflammatory strategies, may exert system-wide effects by reconfiguring network dynamics rather than correcting individual pathways. This perspective suggests that biomarker-stratified, network-calibrated interventions may offer a broader systems-level therapeutic rationale than single-pathway approaches.

## 1. Introduction

Aging is the dominant risk factor for most chronic diseases, yet its biological organization remains difficult to reconcile across molecular, cellular, and systemic scales. Existing frameworks, including the hallmarks of aging [1,2] and the geroscience hypothesis [3], have catalogued key mechanistic processes [4,5,6], but have not explained how these processes interact to produce organism-level decline and multimorbidity.

A central limitation of prevailing models is their predominantly modular structure. Although individual mechanisms such as genomic instability, mitochondrial dysfunction, and chronic inflammation are well characterized, their integration is often treated descriptively rather than mechanistically. Systems biology and network medicine emphasize that physiological states emerge from interactions among interconnected components rather than isolated lesions [7,8,9], yet existing frameworks describe the hallmarks without explaining how they converge into organism-level decline.

Here, a complementary framework is proposed in which aging is understood as progressive destabilization of interacting regulatory networks. Physiological function depends not only on the integrity of individual components but on the stability of their interactions across scales. Aging therefore reflects a progressive loss of network coordination, manifest empirically as non-linear accumulation of health deficits [10,11,12,13] and the structured clustering of chronic conditions characteristic of multimorbidity [14].

**Figure 1:**
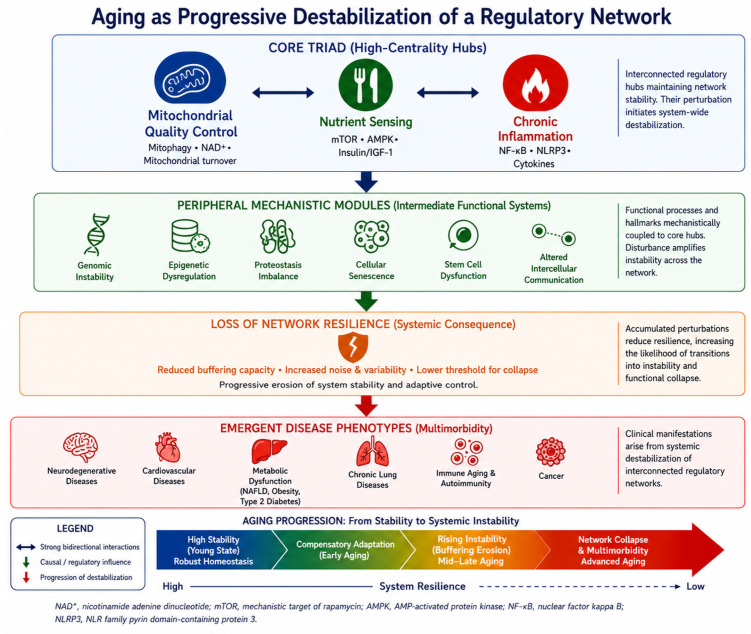
Aging as progressive destabilization of a regulatory network. The figure illustrates aging as a systems-level process organized into four hierarchical tiers. The Core Triad comprises three putative high-centrality hubs: mitochondrial quality control, nutrient sensing, and chronic inflammation. These interact through strong bidirectional regulatory connections and collectively maintain network stability; their perturbation is proposed to initiate system-wide destabilization. Peripheral Mechanistic Modules represent intermediate functional systems, including genomic instability, epigenetic dysregulation, proteostasis imbalance, cellular senescence, stem cell dysfunction, and altered intercellular communication, which are mechanistically coupled to the core triad through which network perturbations are proposed to propagate across the network. Loss of Network Resilience represents a systemic consequence of accumulated perturbation, characterized by reduced buffering capacity, increased noise and variability, and a progressively lower threshold for functional collapse. Emergent Disease Phenotypes represent the clinical manifestations proposed to arise from this systemic destabilization, including neurodegenerative diseases, cardiovascular diseases, metabolic dysfunction (including non-alcoholic fatty liver disease, obesity, and type 2 diabetes), chronic lung diseases, immune aging and autoimmunity, and cancer. The horizontal arrow at the bottom of the figure depicts the progression of aging from high stability (robust homeostasis in the young state) through compensatory adaptation, rising instability with buffering erosion, to network collapse and multimorbidity in advanced aging, accompanied by a corresponding decline in system resilience. Arrows are limited to three types: bidirectional arrows indicate strong reciprocal interactions among core triad components; single-headed arrows indicate causal or regulatory influence from one tier to the next; and downward arrows indicate progression of destabilization through the hierarchy. NAD^+^, nicotinamide adenine dinucleotide; mTOR, mechanistic target of rapamycin; AMPK, AMP-activated protein kinase; NF-κB, nuclear factor kappa B; NLRP3, NLR family pyrin domain-containing protein 3. The network structure depicted is conceptual and heuristic, derived from convergence across experimental and translational literature; it does not represent a formally validated network topology.

Within this architecture, mitochondrial quality control, nutrient sensing, and chronic inflammation form a proposed high-centrality regulatory core. These three modules are designated putative dominant hubs on the basis of their pleiotropic connectivity in the literature reviewed here: each is reported to directly regulate or be regulated by the majority of remaining hallmarks, a pattern consistent with, though not formally established as, disproportionate destabilizing potential relative to more peripheral nodes (Figure 1). Mitochondria act as signaling hubs coordinating redox balance and transcriptional adaptation [15,16], while age-associated alterations in mitophagy, mitochondrial turnover, and NAD^+^ (nicotinamide adenine dinucleotide)-dependent signaling extend across regulatory layers [15,17,18,19,20,21]. In parallel, inflammatory signaling both responds to and reinforces mitochondrial dysfunction, including through activation of the NLRP3 (NLR family pyrin domain-containing protein 3) inflammasome [22,23,24], establishing bidirectional coupling between metabolic stress and immune activation [25].

This framework extends existing models in three key ways. First, it reframes network stability—rather than individual hallmark integrity—as the primary determinant of aging dynamics. Second, it identifies mitochondrial regulation as the operational hub linking metabolic and inflammatory layers. Third, it incorporates non-linear systems properties: feedback loops, bistability, and threshold effects—as exemplified by mitohormesis [26,27]—mean that identical perturbations produce qualitatively different outcomes depending on network state. This has direct implications for therapeutic calibration and for individual variability in aging trajectories. The triangular functional core proposed here should be regarded as a heuristic organizing framework derived from convergence across experimental and translational literature, rather than as a formally validated network topology. This distinction is addressed explicitly in the Limitations section. This article constitutes a narrative and conceptual review. The literature was identified through searches of PubMed and Web of Science conducted iteratively between May and June 2026 using terms including aging, network medicine, mitochondrial dysfunction, NAD^+^ metabolism, NLRP3 inflammasome, hallmarks of aging, and multimorbidity, with no date restriction on publication year. In practice, priority was given to studies reporting a direct molecular or cellular pathway linking a candidate process to mitochondrial quality control, nutrient sensing, or chronic inflammatory signaling, and when findings extended beyond a single model system to human tissue, human biomarker data, or clinical outcomes. This was not applied as a formal a priori protocol but reflects, in retrospect, the basis on which sources were selected; in descending order, interventional human trials, human biomarker-level or observational data, and mechanistic preclinical studies were favored, with primary research articles and seminal reviews prioritized over secondary commentary. Studies focusing exclusively on single molecular targets without broader network or systemic implications, and animal studies lacking translational relevance to human aging biology, were generally not prioritized.

## 2. Integrative Biology as a Multi-Scale Framework

Molecular mechanisms that are well-characterized in isolation routinely fail to predict organism-level phenotypes. This is not a gap in mechanistic knowledge—it reflects the emergent nature of biological networks. Network medicine conceptualizes disease as a perturbation of functional modules rather than isolated lesions [7,8]. Multi-scale integration, as used here, denotes the capacity of molecular events to propagate through cellular, tissue, and systemic layers, a property absent from pathway-level models but essential for understanding how localized dysfunction generates systemic disease.

Fusion and fission regulate organelle integrity and metabolic adaptability [17,28,29]. Mitophagy selectively removes damaged mitochondria, constraining propagation of oxidative and inflammatory stress signals [15,16,17,30,31,32]. Mitochondrial stress also activates the UPRmt, linking organelle dysfunction to nuclear transcriptional remodeling [15,17,18].

NAD^+^-dependent signaling, including SIRT1-mediated deacetylation of PGC-1α, couples bioenergetics to mitochondrial biogenesis and broader transcriptional control [15,19,20,21], and age-related mitochondrial decline in humans is associated with insulin resistance [33,34,35], a pattern consistent with, though not sufficient on its own to establish, systemic metabolic coupling.

Mitochondrial dysfunction activates the NLRP3 inflammasome [22], creating bidirectional feedback between metabolic stress and immune activation [36,37] that amplifies both. Mitochondria, nutrient-sensing pathways, and inflammatory signaling thus constitute a densely interconnected triad whose collective perturbation—rather than the failure of any single component—underlies the systemic character of biological aging (Figure 1). Within this triad, mitochondria are labelled an operational hub because they execute the downstream bioenergetic and redox consequences of upstream signals; nutrient sensing is labelled a regulatory integrator because it directly senses and adjusts mitochondrial and inflammatory activity in response to metabolic state; and chronic inflammation is labelled an amplifier hub because it converts localized mitochondrial or metabolic perturbation into systemic, self-reinforcing signalling.

## 3. Hallmarks of Aging as an Interconnected Network

The hallmarks of aging can be reinterpreted as dynamically interacting network nodes rather than independent processes [1,2]. Genomic and epigenomic regulation is tightly coupled to metabolic state through NAD^+^-dependent enzymes and mitonuclear signaling [19,20,38,39,40]. PARP1 (poly (ADP-ribose) polymerase 1) and sirtuins compete for the same NAD^+^ pool—the former during DNA damage repair, the latter during transcriptional regulation. NAD^+^ depletion thus creates direct metabolic coupling between genome maintenance and mitochondrial signaling, linking two hallmarks otherwise treated as independent. Consequently, genome maintenance and epigenetic remodeling are inseparable from cellular bioenergetics and mitochondrial signaling.

Loss of proteostasis intersects with mitochondrial quality control, as reduced mitophagy permits accumulation of dysfunctional organelles that amplify oxidative and inflammatory stress [16,22]. Reduced mitophagy thus bridges proteostatic collapse, metabolic inflexibility, and immune activation within a single mechanistic chain. Deregulated nutrient sensing further modulates autophagy and mitochondrial biogenesis; pharmacological inhibition of mTOR extends lifespan in model organisms [41], underscoring the systemic influence of nutrient-sensing nodes.

Cellular senescence contributes to inflammatory amplification through the SASP (senescence-associated secretory phenotype), which in turn impairs mitochondrial function in neighboring cells and induces paracrine senescence, establishing a self-reinforcing cycle that progressively expands network instability [1,2,42]. Senolytic strategies attenuate age-related phenotypes in preclinical models [43], supporting the role of senescence as a network amplifier rather than a static cellular endpoint. In parallel, altered intercellular communication and stem cell exhaustion reflect downstream consequences of chronic inflammatory signaling and metabolic constraints, reinforcing the view that hallmarks operate as an interacting system rather than as a list of parallel pathways. The hallmarks do not merely co-occur—they are coupled, and perturbation of any one propagates through the network according to connectivity, not causal proximity (Table 1).

## 4. Mitochondrial Regulation of Network Stability

Mitochondria occupy a uniquely central position in the aging network [44,45]. No other organelle simultaneously governs energy metabolism, redox signaling, apoptosis, innate immune activation, and epigenetic reprogramming—a functional breadth that makes mitochondrial dysfunction inherently propagative across regulatory modules. Balanced fusion–fission dynamics maintain structural integrity and metabolic flexibility [17,28,29], while impaired dynamics contribute to cardiovascular dysfunction and reduced stress tolerance [46,47]. Mitochondrial plasticity is therefore not a secondary feature but a determinant of systemic stability.

Reduced mitophagy efficiency permits accumulation of dysfunctional mitochondria, amplifying redox imbalance and inflammatory signaling [15,16,17,22,30,31,32]. Because mitophagy constrains propagation of mitochondrial stress signals, its decline effectively increases noise within cellular regulatory circuits and reduces buffering capacity. Coordination between mitophagy and mitochondrial biogenesis is required to maintain organelle homeostasis [16,48,49], and disruption of this balance accelerates functional decline.

Age-associated NAD^+^ decline impairs mitonuclear communication, decoupling energetic state from transcriptional and stress-response programs [15,19,20,21,38]—a shift that propagates mitochondrial dysfunction into proteostatic and immune modules. Adaptive signaling pathways such as the UPRmt further link organelle stress to nuclear transcriptional remodeling [18].

Mitohormesis illustrates non-linear redox regulation, whereby moderate mitochondrial stress can induce adaptive resilience, whereas excessive stress promotes dysfunction [26,27]. A key mediator of this non-linearity is mitochondrial ROS production. At physiological levels, ROS act as redox signalling molecules that regulate adaptive responses; sustained overproduction drives oxidative damage and accelerates hallmark progression. Comparative analyses across species show that mitochondrial H_2_O_2_ production rate per unit oxygen consumed correlates inversely with maximum lifespan across species, positioning mitochondrial ROS dynamics as a quantitative effector of biological aging [50]. Effective interventions must therefore calibrate redox signalling dynamics rather than indiscriminately suppress oxidative pathways. Exercise and caloric restriction are the most robustly validated interventions for network recalibration: both activate mitohormetic programmes, improve mitochondrial quality control, reduce inflammatory tone, and extend healthspan across multiple model systems. Low-dose mitochondrial uncouplers can partially mimic these responses, but the breadth and durability of exercise- and caloric restriction-induced effects remain unmatched by pharmacological approaches.

Secondary mitochondrial dysfunction, defined here as mitochondrial impairment arising downstream of other processes such as inflammation, metabolic dysregulation, or proteostatic stress, rather than from primary defects in mitochondrial or mtDNA-encoded components, is widely observed in metabolic and cardiovascular disease [33,34,35,44,46,51,52,53]. Mitochondrial dysfunction therefore occupies a topologically privileged position in the aging network. As the node through which metabolic, proteostatic, and inflammatory signals converge, its progressive failure reconfigures the entire regulatory landscape rather than disrupting a single functional domain. At the cellular level, this progression follows a recognizable pathological sequence: impaired mitochondrial quality control increases steady-state ROS exposure and oxidative macromolecular damage, which activates the NLRP3 inflammasome and drives a senescence-associated secretory phenotype (SASP); the resulting chronic cytokine output promotes extracellular matrix remodeling and fibrotic change in affected tissues, ultimately manifesting as organ-level functional decline and the degenerative disease phenotypes discussed in Section 5.

## 5. Age-Related Diseases as Emergent Network Phenotypes

Neurodegenerative diseases exhibit mitochondrial dysfunction, impaired mitophagy, and inflammatory activation [54,55]. In Alzheimer’s disease, mitochondrial bioenergetic deficits and altered dynamics precede overt neuronal loss [54,55], suggesting that metabolic instability contributes to disease initiation rather than merely reflecting downstream damage. Deregulated nutrient sensing amplifies this vulnerability: mTOR hyperactivation impairs autophagy and mitophagy, while AMPK hypoactivation reduces metabolic flexibility. Activation of the NLRP3 inflammasome links mitochondrial stress to neuroinflammation [23], and chronic cytokine release from activated microglia creates a self-reinforcing inflammatory loop that further compromises neuronal bioenergetics [56]. This temporal precedence has mechanistic weight: it positions mitochondrial dysfunction as a driver of neurodegeneration rather than a consequence—an important distinction for identifying early intervention windows.

Cardiovascular aging involves impaired mitochondrial energetics, altered substrate utilization, and increased inflammatory signaling [33,46,47,57]. Reduced mitochondrial flexibility compromises cardiac stress responses, while chronic low-grade inflammation promotes vascular remodeling and plaque instability [36,58]. Deregulated nutrient sensing further impairs cardiac autophagy through mTOR activation and reduces metabolic flexibility through AMPK suppression, limiting the heart’s response to energetic stress. The clinical efficacy of anti-inflammatory therapy in reducing cardiovascular events [59] supports the interpretation that inflammatory signaling operates as a modifiable high-centrality node within aging-associated networks.

Type 2 diabetes reflects integrated mitochondrial, metabolic, and inflammatory disturbances [33,35,51,60]. Mitochondrial dysfunction contributes to insulin resistance, while chronic inflammation exacerbates metabolic dysregulation. These interdependencies exemplify the network destabilization model: in type 2 diabetes, no single causal pathway is sufficient to explain the full phenotype, and effective intervention may require simultaneous modulation of multiple high-centrality nodes rather than sequential correction of individual pathways.

Cancer represents a distinct pathological network outcome within this systems framework: it engages the same core nodes highlighted here, including mTOR signaling, mitochondrial metabolism, and NF-κB-related inflammation, but channels their dysregulation toward adaptive rewiring of metabolic circuitry rather than network collapse, exploiting the reduced regulatory precision characteristic of aged tissue [61,62]. Functional mitochondria support biosynthetic demands and redox balance in tumor cells [62,63], illustrating how oncogenesis actively co-opts the same metabolic nodes that aging-associated instability otherwise fails to suppress. The distinction between permissive instability and functional collapse highlights the context-dependence of network-level outcomes.

Multimorbidity thus reflects overlapping network failures rather than coincidental co-occurrence of independent diseases. As regulatory hubs fail, the probability of multiple simultaneous disease phenotypes increases according to network topology, not pathological independence—a prediction with direct implications for how multi-morbid patients are stratified and treated.

## 6. Network-Oriented Therapeutic Modulation

The network destabilization model has a direct therapeutic implication: interventions should target nodes with the highest systemic connectivity, where modulation propagates across regulatory layers rather than correcting isolated deficits. Interventions targeting nutrient-sensing pathways represent one of the most extensively studied strategies in this regard. Pharmacological inhibition of mTOR extends lifespan in multiple model organisms [41,64,65,66,67] and modulates immune function in older adults [41,68,69]. Rather than acting through a single downstream pathway, mTOR inhibition influences autophagy, mitochondrial turnover, protein synthesis, and metabolic adaptation, illustrating how modulation of a high-centrality node can propagate across regulatory layers. This pleiotropic reach carries a calibration requirement: mild mTOR inhibition promotes clearance, but excessive suppression impairs tissue repair—a dose-sensitivity the network framework explicitly predicts.

Metformin similarly targets nutrient sensing and mitochondrial metabolism through activation of AMP-activated protein kinase (AMPK) and indirect mTOR modulation [69,70]. Its established cardiometabolic benefits in type 2 diabetes highlight the translational potential of metabolic reprogramming, although long-term effects on multimorbidity and global resilience remain under investigation.

Mitochondrial-targeted strategies focus on restoring organelle quality and signaling competence. Urolithin A has been shown to induce mitophagy in model organisms [71] and to improve mitochondrial biomarkers in humans [72], while NAD^+^ precursors increase systemic NAD^+^ levels and modulate metabolic parameters in humans [73,74,75,76,77], an effect so far established only at the biomarker level, with clinical endpoints such as reduced multimorbidity or extended healthspan not yet demonstrated. Spermidine extends lifespan and reduces cardiovascular risk through autophagy-dependent mechanisms [78,79]: it inhibits EP300 acetyltransferase, triggering histone hypoacetylation and autophagy induction [80], and activates mitophagy to restore mitochondrial quality. These effects reduce NLRP3 inflammasome activation and lower circulating inflammatory markers. Dietary spermidine intake is associated with reduced mortality, improved cardiac outcomes, and signals of cognitive benefit [79,81,82], further supporting the importance of mitochondrial and proteostatic integration.

Cellular senescence represents another network amplifier amenable to modulation. Senolytics provide a direct test of the network hypothesis: if senescent cells amplify instability via the SASP, their elimination should produce system-wide effects disproportionate to cell numbers removed—a prediction supported preclinically [43,83,84] and now entering human trials. Anti-inflammatory therapy targeting IL-1β (interleukin-1β) has also demonstrated a reduction in cardiovascular events [59], providing proof-of-principle that inflammatory nodes are clinically modifiable. Alpha-ketoglutarate (AKG), an endogenous TCA cycle intermediate whose levels decline with age, extends lifespan and compresses morbidity in mice while suppressing chronic inflammatory cytokines [85,86]; human evidence remains limited to an early-phase clinical trial protocol, and the mouse findings should not yet be assumed to translate to comparable effects in humans. Its mechanisms are multi-nodal: AKG is an obligate cofactor for TET DNA demethylases and Jumonji C-domain histone demethylases, linking mitochondrial metabolic state to epigenetic gene regulation; it also suppresses NF-κB-mediated inflammation through prolyl hydroxylase-dependent inhibition of IKKβ [87]. Together, these effects span mitochondrial function, epigenetic regulation, and chronic inflammation. Pyrroloquinoline quinone (PQQ), a redox-active o-quinone compound found in diverse foods, stimulates mitochondrial biogenesis through SIRT1/PGC-1α activation and attenuates inflammatory markers [88,89], supporting its candidacy as a multi-node intervention targeting both mitochondrial quality control and chronic inflammation; clinical evidence remains exploratory, derived from a single small trial, and requires replication before any clinical recommendation can be made.

Because aging networks are non-linear and context-dependent, interventions targeting high-centrality nodes may require calibrated modulation rather than maximal inhibition. The interventions discussed here are included as mechanistic exemplars illustrating modulation of distinct high-centrality nodes rather than as an exhaustive therapeutic inventory. A summary is provided in Table 2.

## 7. Limitations and Conceptual Boundaries

Although the network destabilization framework offers integrative explanatory power, several limitations require consideration. Biological networks are inherently context-dependent, and the relative centrality of regulatory nodes varies across tissues, genetic backgrounds, and environmental exposures. Aging trajectories display substantial heterogeneity, and network-based analyses of health deficits reveal non-linear accumulation patterns rather than uniform progression. Identical interventions targeting mitochondrial quality, nutrient sensing, or inflammatory signaling may therefore produce divergent effects depending on baseline network configuration and individual resilience. A key conceptual limitation is that the network destabilization framework, as presented here, remains qualitative. The designation of mitochondrial quality control, nutrient sensing, and inflammatory signaling as high-centrality nodes is supported by extensive empirical evidence but has not been formally validated by quantitative network analysis of aging-associated interactomes. Future work employing graph-theoretic methods on multi-omics datasets will be necessary to test whether these nodes meet formal criteria of hub-centrality and to identify additional high-centrality nodes that the present framework may overlook [92,93].

Network dynamics are intrinsically non-linear. Moderate mitochondrial stress may activate adaptive responses consistent with mitohormesis [26,27], whereas excessive or chronic stress promotes dysfunction. Similarly, pharmacological modulation of nutrient-sensing pathways such as mTOR can extend lifespan in model organisms [65], yet excessive suppression may compromise immune competence or tissue repair. Translational limitations further constrain interpretation. NAD^+^ augmentation consistently increases systemic NAD^+^ levels and modulates metabolic parameters [73,74,75,76,77], but whether these changes translate into reduced multimorbidity, improved functional capacity, or extended healthspan remains unestablished. Adequately powered, long-duration trials are needed. Similarly, mTOR modulation influences immune parameters in older adults [68], but durable effects on mortality and multimorbidity remain insufficiently established.

Finally, while the triangular functional core emphasizes mitochondrial quality control, nutrient sensing, and inflammatory signaling as high-centrality hubs, not all age-related pathologies can be reduced exclusively to these modules. The network destabilization framework should therefore complement mechanistic specificity rather than replace it.

## 8. Conceptual Synthesis and Future Directions

The convergence of mitochondrial dysfunction, metabolic inflexibility, and inflammatory amplification across neurodegenerative, cardiovascular, metabolic, and oncological diseases points to shared regulatory architecture rather than disease-specific pathology. These phenotypes arise from the progressive failure of shared hubs, not independent pathological cascades—a perspective that opens new angles for both research design and therapeutic strategy.

Translation of network-based aging biology into clinical practice will likely require rethinking trial design. Traditional randomized trials typically evaluate single agents in heterogeneous populations using static endpoints. Precision geroscience approaches may benefit from stratification based on mitochondrial function, metabolic flexibility, inflammatory tone, or composite resilience indices rather than chronological age alone [94,95]. Incorporation of longitudinal systems-level biomarkers and adaptive trial designs may improve the detection of clinically meaningful effects.

Several testable predictions follow. First, network instability should manifest as correlated changes across three domains: mitochondrial function (NAD^+^/NADH ratio, membrane potential proxies), metabolic flexibility (respiratory quotient variability, ketone body production capacity), and inflammatory tone (IL-6 (interleukin-6), CRP (C-reactive protein), NLRP3 activation markers). Composite indices capturing this inter-domain coupling may outperform single biomarkers in predicting age-related decline. Second, interventions targeting high-centrality nodes may produce system-wide effects that exceed those of pathway-specific modulation, detectable as correlated improvements across multiple hallmark-associated biomarkers rather than isolated endpoint changes. Third, individual variability in aging trajectories may reflect quantifiable differences in baseline network configuration and resilience capacity, suggesting that composite network-state indices could help stratify individuals by biological rather than chronological age and predict differential responses to intervention. Future studies integrating multi-omics data with network analysis may help operationalize these predictions and bring the framework closer to formal quantitative validation. Viewing aging as progressive destabilization of interconnected regulatory networks provides a mechanistic framework linking molecular hallmarks with multimorbidity and may support the development of more integrative therapeutic strategies. Formal validation of this framework will require graph-theoretical analysis of longitudinal multi-omics and clinical datasets to test the centrality, connectivity, and predictive value of the proposed core nodes.

## Figures and Tables

**Table 1:** Relationship of canonical aging hallmarks to the triangular functional core. For each hallmark, the primary functional relationship to mitochondrial quality control, nutrient sensing, and chronic inflammation is indicated, along with the principal mechanistic rationale and key supporting references. Upstream: the hallmark drives dysfunction within the core. Downstream: the hallmark arises as a consequence of core perturbation. Bidirectional: the hallmark both influences and is influenced by core nodes. Emergent: the hallmark arises from convergent interactions across multiple core nodes. This classification is an interpretive synthesis of the literature reviewed here and is not derived from formal network modeling or quantitative centrality analysis.

Hallmark	Relation to Triangular Core	Principal Mechanistic Rationale	Key Ref.
Mitochondrial dysfunction	Core node	Primary operational hub governing energy metabolism, redox signaling, UPRmt, and NLRP3 inflammasome activation	[15,44]
Deregulated nutrient sensing	Core node	mTOR and AMPK are central regulators of mitochondrial biogenesis, autophagy, and metabolic flexibility; directly modulates both mitochondrial quality and inflammatory tone	[41]
Chronic inflammation	Core node	NLRP3 inflammasome, NF-κB, and cytokine networks create bidirectional feedback with mitochondrial dysfunction and nutrient-sensing pathways	[22,23]
Epigenetic alterations	Bidirectional regulator	NAD^+^-dependent sirtuins and DNA methylation are regulated by mitochondrial metabolites, including NAD^+^ and alpha-ketoglutarate (AKG); epigenetic reprogramming feeds back to mitochondrial biogenesis	[19,20,38]
Genomic instability	Upstream trigger; downstream amplifier	PARP1/sirtuin competition for NAD^+^ couples DNA repair to mitochondrial signaling; ROS-driven DNA damage amplifies genomic instability	[38,39]
Loss of proteostasis	Downstream; feedback amplifier	Impaired mitophagy permits accumulation of dysfunctional organelles; proteotoxic stress activates inflammatory signaling and exacerbates metabolic dysregulation	[16,22]
Cellular senescence	Emergent node	SASP is driven by NF-κB activation (inflammatory core) and mitochondrial dysfunction; paracrine senescence propagates instability across tissues	[1,2,42]
Stem cell exhaustion	Downstream	Chronic inflammatory signaling and metabolic constraints impair stem cell self-renewal; mitochondrial dysfunction reduces regenerative capacity	[1,2]
Altered intercellular communication	Downstream; amplifier	Inflammageing and SASP alter systemic cytokine environment; mitochondria-derived DAMPs contribute to non-cell-autonomous signaling	[36,37]

**Table 2:** Therapeutic interventions targeting high-centrality nodes and closely associated downstream processes in the aging network. The table presents a representative, non-exhaustive selection of interventions chosen on the basis of mechanistic relevance to the three core nodes (mitochondrial quality control, nutrient sensing, and chronic inflammation), as well as to mechanistically linked downstream processes (autophagy, cellular senescence) through which several interventions act, and availability of at least preliminary human data. Target Node indicates the regulatory module or closely associated process primarily modulated. Principal Mechanism summarizes the dominant molecular action. Human Evidence reflects the current level of clinical support, ranging from randomized controlled trials (rapamycin/mTOR inhibitors, anti-IL-1β therapy, Coenzyme Q10) to biomarker-level clinical observations (NAD^+^ precursors, urolithin A) and epidemiological associations (spermidine); fisetin evidence remains predominantly preclinical. This classification of target nodes is an interpretive synthesis of the literature reviewed here and is not derived from formal network modeling. Abbreviations: mTOR, mechanistic target of rapamycin; mTORC1, mTOR complex 1; AMPK, AMP-activated protein kinase; NAD^+^, nicotinamide adenine dinucleotide; NF-κB, nuclear factor kappa B; NLRP3, NLR family pyrin domain-containing protein 3.

Intervention	Target Node	Principal Mechanism	Human Evidence	Key Ref.
Rapamycin/mTOR inhibitors	Nutrient sensing (mTOR)	Inhibition of mTORC1; enhanced autophagy; metabolic reprogramming	Lifespan extension in model organisms; immune modulation in older adults	[65,68]
Metformin	Nutrient sensing/mitochondrial metabolism	AMPK activation; indirect mTOR modulation; reduced hepatic gluconeogenesis	Reduced cardiometabolic risk in type 2 diabetes	[69,70]
NAD^+^ precursors (NR, nicotinamide riboside; NMN, nicotinamide mononucleotide)	Mitonuclear communication/metabolic regulation	Restoration of NAD^+^ levels; improved mitochondrial signaling	Increased NAD^+^ levels; modulation of metabolic biomarkers	[73,74,75,76,77]
Urolithin A	Mitophagy/mitochondrial quality control	Induction of mitophagy; improved mitochondrial turnover	Improved mitochondrial biomarkers and muscle function	[71,72]
Spermidine	Autophagy	Induction of autophagy; enhanced cellular stress resistance	Association with reduced mortality; signals of cognitive benefit	[79,81,82]
Alpha-ketoglutarate (AKG)	Mitochondrial metabolism; nutrient sensing; chronic inflammation	TCA cycle intermediate; mTOR-independent inhibition of inflammatory cytokine signaling; epigenetic regulation via histone demethylases	Lifespan and healthspan extension with morbidity compression in mice; early-stage clinical exploration (ABLE trial)	[85,86]
Pyrroloquinoline quinone (PQQ)	Mitochondrial quality control; chronic inflammation	Stimulation of mitochondrial biogenesis via SIRT1/PGC-1α; attenuation of inflammatory markers	Improvements in mitochondrial biomarkers and cognition in older adults with mild cognitive impairment (RCT)	[88,89]
Fisetin (senolytic)	Cellular senescence/inflammation	Reduction in senescent cell burden; decreased SASP signaling	Preclinical lifespan extension; early-stage human exploration	[90]
Anti-IL-1β therapy	Chronic inflammation	Inhibition of inflammatory signaling (IL-1β)	Reduced cardiovascular events	[59]
Coenzyme Q10	Mitochondrial bioenergetics	Support of the electron transport chain function	Improved outcomes in chronic heart failure	[91]

## Data Availability

No new data were created or analyzed in this study. Data sharing is not applicable.
